# Increase in number of helminth species from Dutch red foxes over a 35-year period

**DOI:** 10.1186/1756-3305-7-166

**Published:** 2014-04-03

**Authors:** Frits Franssen, Rolf Nijsse, Jaap Mulder, Herman Cremers, Cecile Dam, Katsuhisa Takumi, Joke van der Giessen

**Affiliations:** 1 National Institute for Public Health and the Environment, Centre for Infectious Disease Control, P.O. Box 1, Bilthoven, BA 3720, The Netherlands; 2 Faculty of Veterinary Medicine, Department of Infectious Diseases and Immunology, Utrecht University, Utrecht, Netherlands; 3 Bureau Mulder-natuurlijk, Consultant for Animal Ecology, Berkenlaan 28, RN 3737 Groenekan, Netherlands; 4 Dr. H.T.s'Jacoblaan 62, Utrecht, BN 3571, Netherlands; 5 National Institute for Public Health and Environment (RIVM), Centre for Zoonoses and Environmental Microbiology (Z&O), Anthonie van Leeuwenhoeklaan 9, P.O. Box 1, Bilthoven, BA 3720, The Netherlands

**Keywords:** Helminth fauna, Red fox, Biodiversity, Molecular analysis, Echinococcus, Toxocara, Taenia, Alaria

## Abstract

**Background:**

The red fox (*Vulpes vulpes*) is host to a community of zoonotic and other helminth species. Tracking their community structure and dynamics over decades is one way to monitor the long term risk of parasitic infectious diseases relevant to public and veterinary health.

**Methods:**

We identified 17 helminth species from 136 foxes by mucosal scraping, centrifugal sedimentation/flotation and the washing and sieving technique. We applied rarefaction analysis to our samples and compared the resulting curve to the helminth community reported in literature 35 years ago.

**Results:**

Fox helminth species significantly increased in number in the last 35 years (p-value <0.025). *Toxascaris leonina*, *Mesocestoides litteratus*, *Trichuris vulpis* and *Angiostrongylus vasorum* are four new veterinary-relevant species. The zoonotic fox tapeworm (*E. multilocularis*) was found outside the previously described endemic regions in the Netherlands.

**Conclusions:**

Helminth fauna in Dutch red foxes increased in biodiversity over the last three decades.

## Background

Long-term studies on parasite communities of marine and terrestrial wildlife hosts were instrumental to evaluating the influence of natural and anthropogenic factors on environmental changes, especially when sampling series span more than ten years [1-3].

For larger mammals, like the red fox, many cross-sectional studies report on the parasitic helminth fauna [4-13] or focus on limited parasite species [10,12,14-19], but long-term studies are rare [9].

In the 1980's, Borgsteede [4] studied the helminth fauna in foxes from the border region in the eastern part of The Netherlands, collected between February 1978 and May 1979. For ensuing decades, this study has been the sole large scale surveillance of helminth fauna in red foxes in the Netherlands.

A series of additional large scale surveillance in red foxes became reality since the initial detection of *Echinococcus multilocularis* in the Netherlands in 1996 [20]. *E. multilocularis* tends to increase in the fox population over the last decades in Europe [21] and therefore, the European Food Safety Authority (EFSA) recommends monitoring this parasite in foxes, especially at the borders of its distribution area in Europe [22]. Following the initial detection in the Netherlands, *E. multilocularis* in foxes was found to disperse in southern Limburg, but not in the central and western part of the Netherlands [20]. Since the Netherlands are a densely populated country with an average human population density of 497/km^2^[23] and a pet population of around 1.5 million dogs [24], a high density of red foxes (0.5 to 4.0 per square kilometre) might potentially lead to exposure of humans and dogs to zoonotic parasites, like *E. multilocularis*[16].

Here, we compared our recent large-scale surveillance of helminth fauna in the population of red foxes from the border region in the eastern part of The Netherlands with the historic studies more than 35 years ago. We evaluated trends in parasite richness by applying the rarefaction analysis [25,26]. In addition, we discuss the relevance of our findings for public health.

## Methods

### Animals

**Figure 1:**
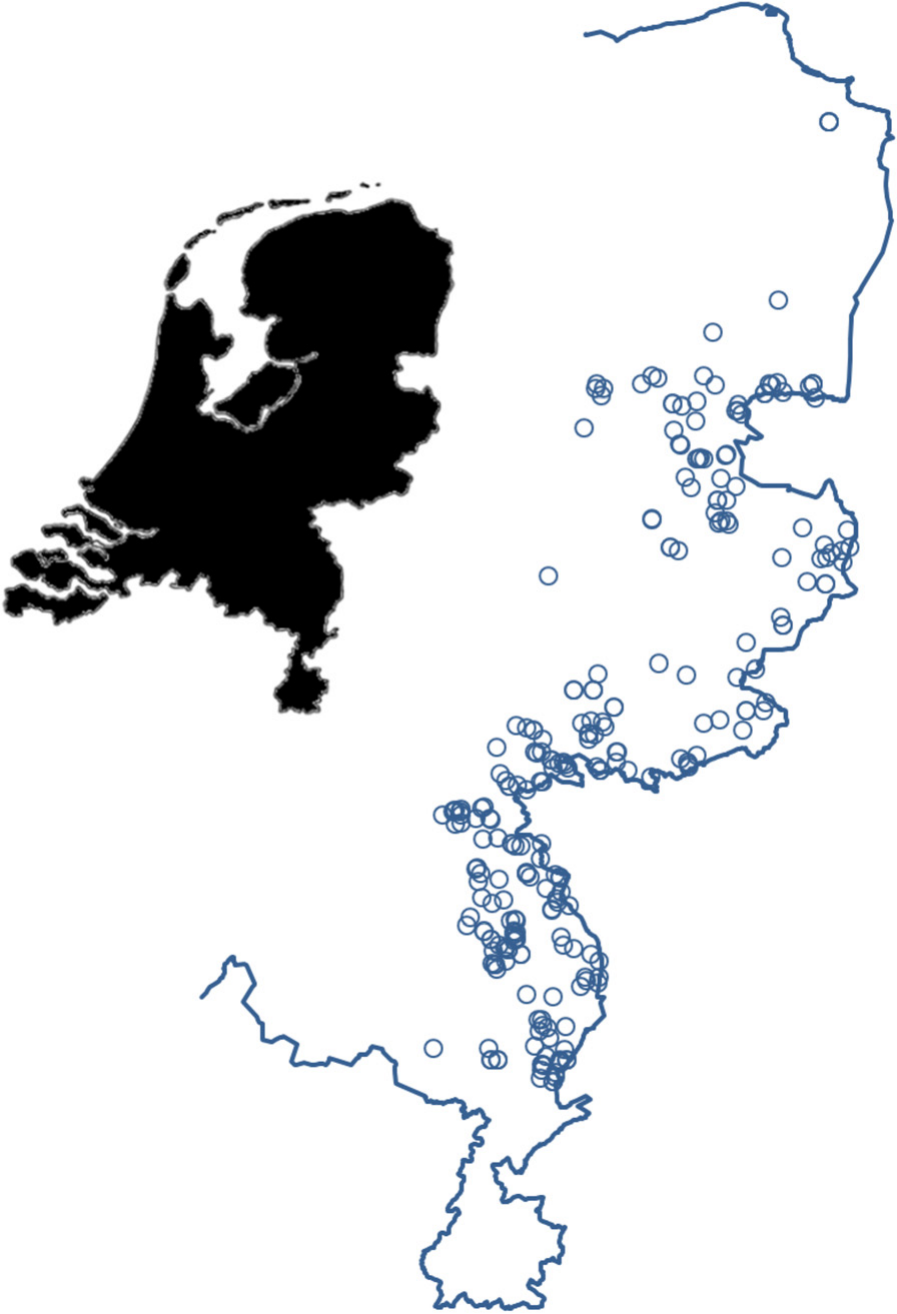
**Geographical origin of individual foxes.** This figure shows the study area along the eastern border of the Netherlands in blue, with a representation of the whole country in black. Circles show the geographical origin of the foxes collected for this study.

From October 2010 until April 2012, routinely shot foxes were collected by hunters and sent to the National Institute for Public Health and Environment (RIVM, Bilthoven, The Netherlands). The chosen fox sample size (288) originated from a strip with a width of 15 km and a length of 266 km at the border with Germany, between Groningen and Limburg (4000 km^2^), excluding the formerly found positive districts (Figure 1). Upon arrival, fox carcasses were stored at −80˚C to inactivate the eggs of *E. multilocularis*[27], according to WHO guidelines [28]. After a minimum period at −80˚C of one week, carcasses were thawed and dissected. Data on weight, measurements, age and gender were collected after thawing. From weight and body size, condition was estimated as the ratio of body weight in grams over body length (nose-anus) in millimetres (body weight/length index, BWL).

The age of the foxes was evaluated by examining tooth wear, especially the wear of the lower incisors and the upper and lower molars and by cutting the root of one or two canines into several 0.15 mm thin slices which were examined microscopically (magnification 20–40 times) under horizontal cross light [29]. Foxes without signs of wear were classified as first year animals [30].

During dissection, the jejunum and faecal material (if present) from the distal colon/rectum of each fox were sampled. The whole small intestines of 262 foxes were evaluated by microscopic examination of mucosal scrapings and macroscopic examination of the opened small intestine. Moreover, distal colon content was used for PCR (see *E. multilocularis-specific PCR identification*); 158 foxes had sufficient faecal content in the colon to be used for additional microscopic analysis after centrifugal sedimentation/flotation.

### Microscopical examination of parasites

#### Small intestine mucosal scraping

The small intestine of each fox was separated and opened. Macroscopically visible helminths were scored and noted. Subsequently, mucosal scrapings were made to screen the mucosal content for small helminths microscopically [31,32]. The presence of intestinal helminths was scored semi-quantitatively: ‘+’ 1–2 individuals, ‘++’ 3–10, ‘+++’ 11–50, ‘++++’ 51–100 and ‘+++++’ >100. Parasites were identified morphometrically and in cases where difficult to identify young adult stages were found, or the freezing/thawing process had damaged the morphology of cestode species, morphological identification was confirmed by PCR (see Molecular identification of parasites). For this purpose, parasite specimens were collected and stored in 70% ethanol until further use.

#### ***Sedimentation***/***flotation of gut content***

When available, about 3 grams of distal colon content were suspended in 50 ml tap water, an 11 ml centrifuge tube was filled with this suspension and the product of centrifugal sedimentation/flotation was examined microscopically. A sucrose solution of 1.28-1.3 g/cm^3^ was used as flotation medium for the faecal examination of eggs and larvae. The centrifugal step for flotation was performed with the cover slip on top of the tube and one slide was examined per sample. The results were scored semi quantitatively using ‘+’ for 1–10 eggs per slide; higher numbers were scored as ’++’ for one to five per microscopic field at 100x (10x10) magnification and ‘+++’ for more than five per microscopic field at the same magnification.

Since fox carcasses were frozen to inactivate zoonotic parasites, the Baermann method could not be used to isolate first stage larvae of *Crenosoma vulpis* and *Angiostrongylus vasorum*. Larvae that were found by CSF, which were not too damaged by the freezing and thawing process were identified morphologically according to McGarry and Morgan [33].

#### ***Screening for cardio***-***pulmonary helminths***

The lungs and hearts of 97 foxes were examined for helminths by opening the right heart and pulmonary arteries up to the level of small branches in the lungs [34]. The bronchi were opened, examined and washed with water, which was sieved through a 150 μm mesh size sieve. The same procedure was used for heart and vessels. Adult and juvenile worms were removed from the sieve and identified morphologically up to species level [35,36].

#### Screening for helminths in the urinary bladder

In addition, four urinary bladders were opened to look for adult worms of *Pearsonema plica*.

### Helminth species number

To evaluate a possible change in helminth species richness, we applied rarefaction analysis [25,26] to the number of distinct helminth species that we identified in 136 foxes. We calculated the rarefaction curve with the software package EstimateS 9.0 [25,26,37] with default settings. Based on the rarefaction curve, we compared our findings with those of historical studies [4-6,8,9].

Foxes, for which biological parameters or geographical data were missing, were excluded from analysis. This limited the available dataset for multifactorial analysis to 136 foxes. For each parasite species, prevalence was calculated and significance of prevalence difference was analyzed with Fisher’s Exact test. Correlations between body condition, age, gender and parasite prevalence were determined by ANOVA (analysis of variance). Fisher’s exact test and ANOVA were performed and the resulting P-values were calculated using Quickcalc (GraphPad Software, Inc. La Jolla, California, USA) and the data analysis module of Microsoft Excel 2007.

#### ***E. multilocularis***-**specific PCR identification**

To analyse the presence of *E. multilocularis* at sub-microscopical level, three grams of colon contents were tested in a single tube nested 12S ribosomal DNA PCR as described previously [20]. PCR products were specified by southern blot hybridization, using *E. multilocularis*- specific probes as described previously [38].

### Molecular identification of parasites

#### DNA isolation and PCR

Parasites were transferred from 70% ethanol and soaked in demineralized water. DNA was isolated using the Qiagen Blood and Tissue Kit (Qiagen NV, Venlo, The Netherlands), according to the manufacturer’s instructions. To confirm the identification of cestode species, a fragment of the mitochondrial cytochrome c oxidase 1 (CO1) gene was amplified as described by Bowles *et al*. [39]. All PCRs were carried out in 50 μl final volume containing 3 μl genomic DNA, 0.5 μl of each forward and reverse primer (50 μM stock) and 25 μl of Qiagen HotstarTaq polymerase master mix (Qiagen NV, Venlo, The Netherlands ). The final reaction volume was adjusted to 50 μl with sterile demineralized water. PCR amplification of the partial CO1 gene was performed using the following conditions: denaturation at 95°C for 15 min, followed by 35 cycles of 1 min denaturation at 95°C, 1 min annealing at 45°C, 1:15 min elongation at 72°C, followed by a final extension step of 7 min at 72°C.

#### DNA sequencing of amplicons

PCR amplicons were purified using standard procedures (ExoSAP-IT®, Affymetrix, Cleveland, Ohio, USA). All DNA sequence PCR reactions were carried out on both DNA strands in 20 μl final volume containing 3 μl of amplicate, 7 μl sequence buffer, 1 μl of Big Dye Terminator and 1 μl of each PCR primer. Sequence PCR was performed under the following conditions: 95°C for 1 min, followed by 25 cycles of 96°C for 10 min, 50°C for 5 min and finally 60°C for 4 min. Trace files of the obtained sequences were generated on an automated ABI sequencer at the Institute’s DNA sequence facility.

#### DNA and phylogenetic analysis

DNA sequences were assembled, edited, and analysed with BioNumerics version 6.6 (Applied Maths NV, Sint-Martens-Latem, Belgium). Obtained CO1 gene sequences were compared to reference sequences present in Genbank after subtraction of the primer sequences. Cluster analysis of the sequences was conducted using the unweighted neighbour-joining algorithm of the BioNumerics program. Bootstrap proportions were calculated by the analysis of 2500 replicates for neighbour-joining trees. Available CO1 sequences of cestodes and trematodes from Genbank were included in the alignment. Sequence homology ≥99% and homology of morphological criteria were considered as proof of identity between isolated and Genbank species.

Unequivocally identified *Alaria alata* isolates from foxes from this study served as out-group in phylogenetic analysis.

## Results

### **Animal age**, **gender and body weight**

In total, 262 foxes were collected. Seventy per cent of the foxes were 7–12 months old at the time of sampling and seven foxes were older than 5 years. This age distribution of shot foxes indicates high hunting pressure as found in previous studies [30,40].

Overall, 55% of the sampled foxes were males and 45% were females, which were evenly distributed over the study area (Figure 1). Males were heavier than females; average body weight / length (BWL) index of males and females differed significantly (ANOVA, P-value < 0.0001). Correlation between BWL index and infection classes was absent for both male (P-value = 0.626) and female foxes (P-value = 0.232).

### Analysis of helminth species number

**Figure 2:**
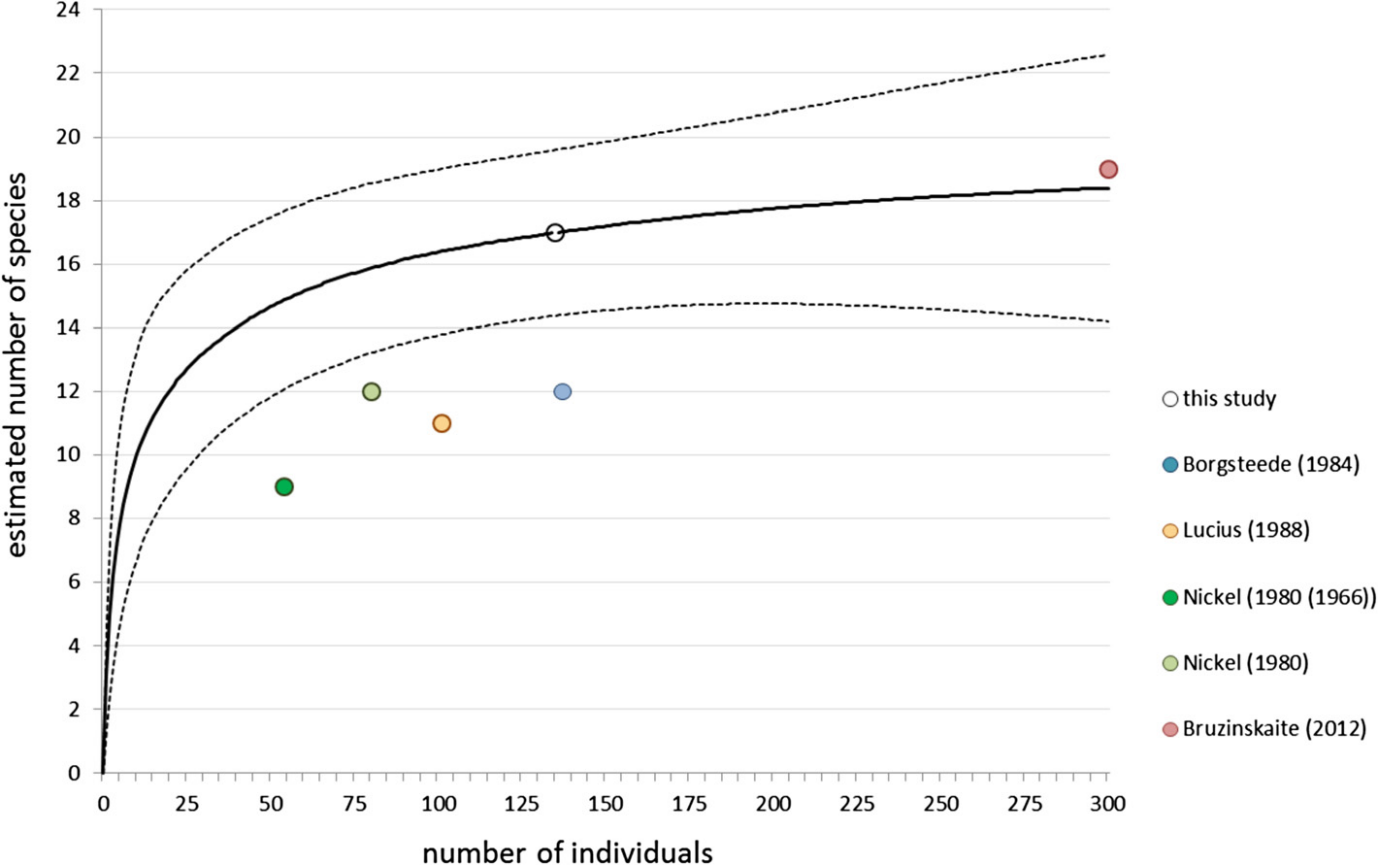
**Analysis of fox parasite species by rarefaction method.** Open circle: the number of distinct parasite species identified from 136 Dutch foxes in this study. Solid circle: the number of distinct parasite species identified from the foxes described in a cited study. Solid line: expected number of distinct parasite species estimated by the rarefaction method based on our data set (i.e. open circle). Dotted line: 95% confidence interval. Nickel *et al*. [9] reported two independent fox populations from different regions, sampled in 1966 (green solid circle) and in 1980 (light green solid circle) respectively.

Seventeen helminth species were identified from our reference data set of 136 foxes. The 95% confidence interval was 14.39 – 19.61 parasite species. The number of parasite species in 137 foxes that were sampled 35 years ago [4] was twelve species, which is a significantly lower species richness (P-value < 0.025) (Figure 2).

### Multiple infections per fox

On average 97.1% of the foxes were infected with one or more out of 17 helminth species, with maximum co-infection levels of eight different species.

**Figure 3:**
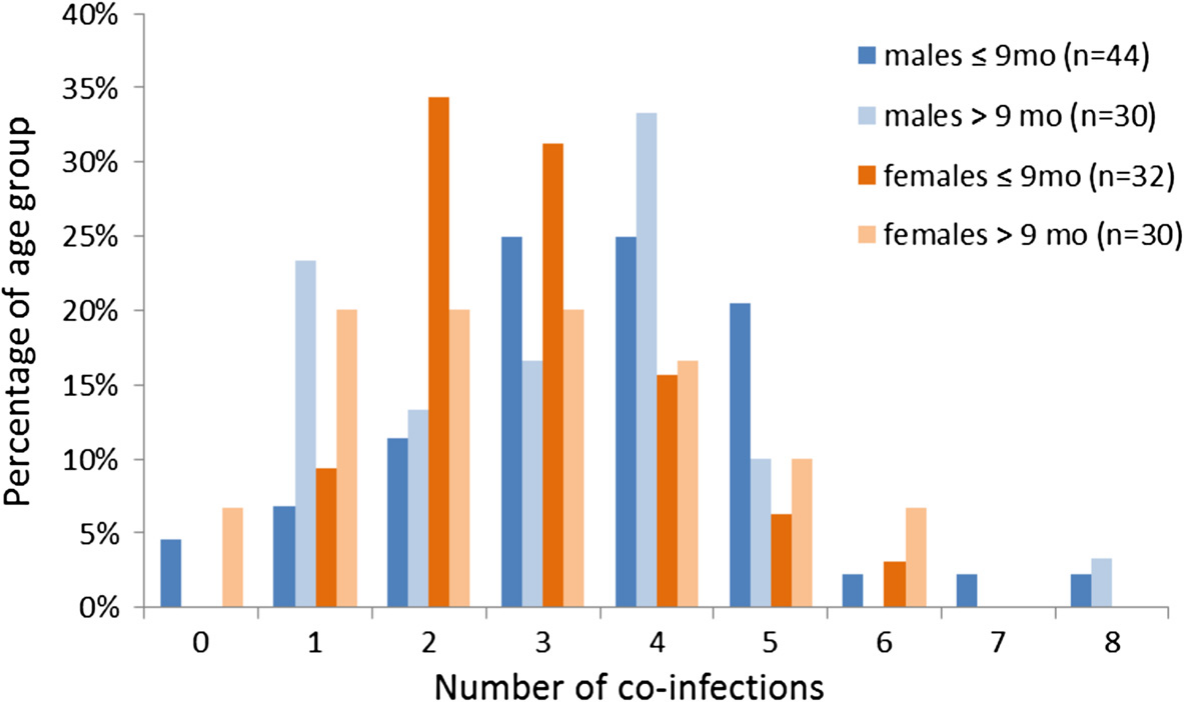
**Number of co**-**infections per age group and per gender.** Male foxes peak at three to four co-infections, females nine months of age and younger peak at two to three co-infections. Male foxes exhibit the highest numbers of co-infection (8). Zero co-infections mean no infection at all. Total number of foxes is 136.

Foxes younger than 10 months were more frequently infected (35-37%) with 2–3 parasite species than foxes older than 10 months (10-27%) (Figure 3).

### Prevalence per helminth species and comparison with other studies

Parasite prevalence was higher in male foxes for the majority of the parasite species (Table 1), although this was only significant for *Toxocara canis* (Fisher’s Exact test, P = 0.013). *T. canis* and *U. stenocephala* were the most prevalent intestinal fox parasites in our study, like in other Western European countries [5-7,14-16]. The prevalences of *T. canis* and *Taenia* spp. were significantly lower in this study compared to the earlier study of Borgsteede [4] (Table 2).

**Table 1:** Overview of parasitic helminths found in Dutch red fox

	**Males(n = 73)**		**Females(n = 63)**		**Overall(n = 136)**		**Means of infection**	**Method**
**Intestinal nematodes**	%	n	%	n	%	n		
1. *Toxocara canis*^*1*^	71.2	52	49.2	31	61.0	83	worm eggs, paratenic hosts	D, CSF
2. *Toxascaris leonina*	1.4	1	3.2	2	2.2	3	worm eggs, paratenic hosts	CSF
3. *Trichuris vulpis*^*2*^	20.5	15	12.7	8	16.9	23	worm eggs	CSF
4. *Uncinaria stenocephala*	60.3	44	47.6	30	54.4	74	free larvae, paratenic hots	CSF, MS
5. *Strongyloides* sp.	9.6	7	20.6	13	14.7	20	free larvae	CSF, MS
**Other nematodes**								
6. *Eucoleus aerophilus* (n = 96)	71.4	35	63.8	30	67.7	65	earthworms, worm eggs	WS
7. *Pearsonema plica*	(2/2)		(2/2)		(4/4)*		worm eggs	D. WS
8. *Capillaria* spp.^3^	52.1	38	47.6	30	50.0	68	worm eggs	CSF
9. *Angiostrongylus vasorum* (n = 96)	6.1	3	2.1	1	4.2	4	terrestrial gastropods, frogs	WS, CSF
10. *Crenosoma vulpis* (n = 96)	24.5	12	8.5	4	16.7	16	terrestrial gastropods	WS, CSF
**Intestinal cestodes**								
11. *Taenia crassiceps*	21.9	16	22.2	14	22.1	30	rodents, lagomorpha	D, MS, PCR
12. *Taenia polyacantha*								
13. *Mesocestoides litteratus*	6.8	5	4.8	3	5.9	8	frogs, intermediate hosts	D, MS, PCR
14. *Echinococcus multilocularis*	1.4	1	0.0	0	0.7	1	rodents, lagomorpha	PCR
**Intestinal trematodes**								
15. *Cryptocotyle lingua*	4.1	3	3.2	2	3.7	5	fish	MS
16. *Isthmiophora melis*	1.4	1	0.0	0	0.7	1	tadpoles	MS
17. *Alaria alata*	17.8	13	15.9	10	16.9	23	tadpoles, frogs	MS, PCR

^1^The observed prevalence in *T. canis* between male and female foxes is significantly different (Fisher’s Exact test, P = 0.013). ^2^This diagnosis was not confirmed by demonstrating adult worms in the colon. ^3^*Capillaria* spp. eggs were not identified to species level due to morphological changes as a result of freezing and thawing. Methods used for detection and speciation. D: dissection, CSF: centrifugal sedimentation/flotation, MS: mucosal scraping, WS: washing and sieving. Species number 6, 9 and 10 were obtained from heart and lung washings for which 96 foxes were available. *: Four out of four urine bladders were found positive for this species, but prevalence was not extrapolated from this limited number of analyses.

**Table 2:** Parasite prevalence in red fox compared to 35 years ago

	**Zoonotic**	**Netherlands**	**Netherlands**	**Fisher Exact**
	**Species**	**Borgsteede(1984)**	**This study**	**P(2-sided)**
		**(n = 137)**	**(n = 136)**	
**Intestinal nematodes**		%	%	
*Toxocara canis*	Yes	73.7	61.0	0.028
*Toxascaris leonina*	No	0	2.2	0.122
*Trichuris* sp.	No	0	16.9	<0.0001
*Uncinaria stenocephala*	Yes	59.9	54.4	0.393
*Strongyloides* sp.	Yes^1^	0.7	14.7	<0.0001
**other nematodes**				
*Eucoleus aerophilus*	No	46.8	67.7	0.285
*Pearsonema plica*	No	23.5	(4/4)^2^	-
*Capillaria spp*.			50.0	-
*Angiostrongylus vasorum* adults/larvae	No	(0)^3^	4.2	0.028
*Crenosoma vulpis* adults/larvae	No	4.5	16.7	0.008
**Cestodes**				
*Taenia* spp.^4^	Yes^5^	53.3	22.1	<0.0001
*Mesocestoides* sp.	No	0	5.9	0.003
*Echinococcus multilocularis*	Yes	0	0.7	0.498
**Trematodes**				
*Cryptocotyle lingua*	No	3.6	3.7	1
*Eupariphium melis*	No	1.5	0.7	1
*Alaria alata*	No	10.9	16.9	0.166
*Opistorchis felineus*	Yes	0	0	-
*Apophallus donicus*	No	0.7	0	0.498
noninfected (over-all)		2.9	2.9	

Differences between this study and the Borgsteede study [4] are indicated (Fisher’s exact test). ^1^*Strongyloides* species are non-zoonotic, whereas *S. stercoralis* is infectious to humans and is a species of warm geographical zones, although found in a dog kennel in Finland [55]. ^2^This species was present in four analysed urinary bladders, therefore prevalence difference was not analysed. ^3^The first documented cases of autochthonous French heartworm were seen in 2009. ^4^Data on *Taenia* species were combined to facilitate comparison with other studies. ^5^In our study, *T. crassiceps* and *T. polyacantha* were found, the former of which is zoonotic.

The combined prevalence of *Toxocara canis* and *Toxascaris leonina* reported in Belgian foxes in 2005 [16] was not different (Fisher’s Exact test, P = 0.315) from the prevalence in our study. The prevalence of *T. canis* in Danish foxes in 2006 [6] was 59.4%, which is almost identical to the level found in this present study, as was the case for *Taenia* species. In contrast, the prevalence of *Uncinaria stenocephala* was significantly higher in Denmark [6], compared to either our data (Fisher’s Exact test, P = 0.0018), historical data from northern Germany [5] (Fisher’s Exact test, P = 0.002), or historical data from the Netherlands [4] (Fisher’s Exact test, P = 0.054).

The prevalences of *Strongyloides* sp., *Eucoleus aerophilus* and *Crenosoma vulpis* was significantly higher than reported in 1984 [4] (Table 2). *Trichuris vulpis*, *Angiostrongylus vasorum*, *Mesocestoides litteratus* and *Echinococcus multilocularis* were new species in the studied area. The trematode *Apophallus donicus*, of which one individual was found by Borgsteede [4] was not identified in the present study. This was also the case for *Hymenolepis* spp., for which rodents are definitive hosts. Adult *Hymenolepids* are regarded as passing species from prey, as is *Molineus patens*, and these were thus excluded from analysis of helminth species parasitic to red fox.

### ***E. Multilocularis***-**specific PCR identification**

All foxes were negative for this species by microscopical examination of mucosal scrapings, but one fox out of 262 investigated foxes was positive for *E. multilocularis* (prevalence 0.7%; 95% CI 0.02-2.1%), using the 12S single tube nested PCR and subsequent southern blot analysis on faecal content. This positive result was confirmed after repeated testing of the faecal content. Up to this study, no positive foxes were identified in the presently studied area.

### Molecular characterisation of intestinal parasites

PCR products of *Taenia polyacantha*, *Taenia crassiceps* and *Alaria alata* were all 403 bp in length. These DNA sequences were submitted to Genbank [accession numbers KF751222-KF751223 (*T. crassiceps*, isolates V1382 and V1336), KF751225-KF751226 (*T. polyacantha*, V1361 and V1269) and KF751233-KF751234 (*A. alata*, V1338 and V1359)].

**Figure 4:**
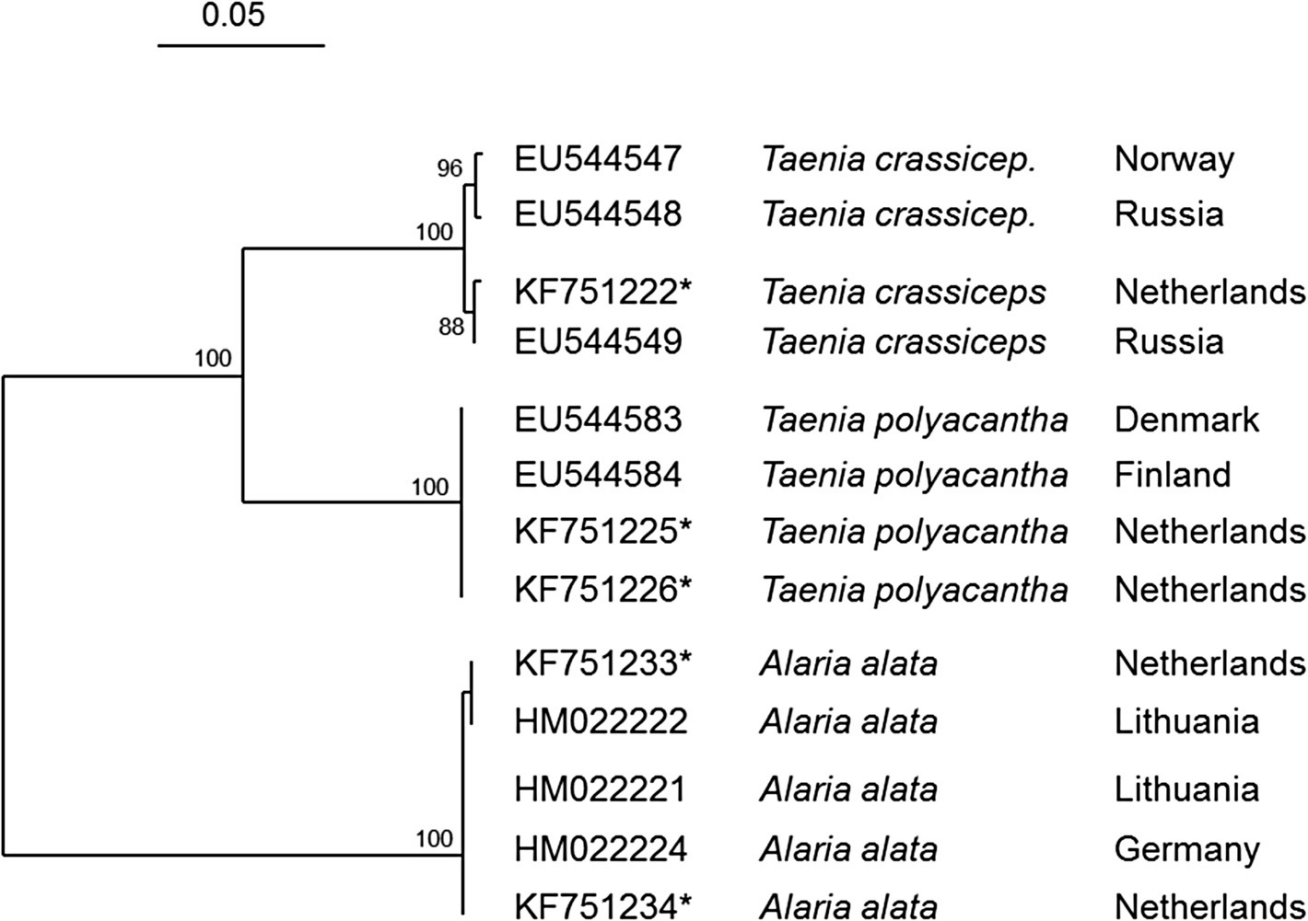
**CO1 Neighbour Joining Tree of European fox cestode isolates.***Taenia* species found in red fox (* this study) show high homology with other European isolates found in Genbank (bootstrap values of 2500 simulations). *Alaria alata* is used as outgroup and here too, the Dutch isolates show high homology with other European isolates from Genbank. Bar indicates base substitutions per site.

Microscopic identification of cestodes was confirmed by cluster analysis of the partial CO1 gene sequences. The inferred Neighbour Joining tree shows very high homology between obtained CO1 sequences and Genbank entries for *T. crassiceps* from Russia and Norway (EU544548, EU544547), *T. polyacantha* from Denmark and Finland (EU544583, EU544584) and for the trematode *A. alata* from Lithuania and Germany (HM022221, HM022222 and HM022224), the latter of which served as outgroup (Figure 4).

## Discussion

This study shows an increased diversity in the helminth parasite community of Dutch red foxes compared to a study conducted in the same region 35 years ago [4]. We report four new records of veterinary importance: *Toxascaris leonina*, *Mesocestoides litteratus*, *Trichuris vulpis* and *Angiostrongylus vasorum*. The finding of a fifth (zoonotic) species –*Echinococcus multilocularis*– has been described earlier for the Netherlands [20], but not in this same geographical area.

We used a combination of microscopic and molecular techniques to evaluate the helminth fauna of red fox as described above, whereas Borgsteede [4] and Lucius *et al*. [5] used microscopy following the washing and sieving technique. Use of the more sensitive PCR technique in this present study might have biased the observed biodiversity to some extent, since it was not available in the period of the study of Borgsteede [4], but this does not explain the observed biodiversity increase compared to older studies. Confirmation of the identity of cestode species that had been found microscopically by PCR in this present study, did not lead to more cestode species compared to historic data. Moreover, even without *E. multilocularis*, which was demonstrated only by PCR, significantly more helminth species were found in this present study, compared to historical data (result not shown). The introduction of *E. multilocularis* and *A. vasorum* into the Netherlands is documented [20,38,41]; these independent studies support the increased biodiversity of helminth fauna in the population of red foxes in the Netherlands. The study of van der Giessen *et al*. [20], for which a combination of mucosal scraping and PCR was used, demonstrated presence of *E. multilocularis* in the eastern border region, both north and south to the present study area, but not in the latter, which was included in that study as well. This finding confirmed the observation of Borgsteede [4] at that time.

Parasites indicated as *Capillaria* spp. might include more fox specific species, like *Eucoleus boehmi*, which is endemic to the Netherlands (H. Cremers, unpublished data), and other species passing through the gut after predation; however these were not further identified to species level.

Rarefaction and extrapolation of parasite richness and abundance data (this study) revealed a significant increase of species richness compared to 12 different fox parasite species determined by Borgsteede [4], 11 species found by Lucius *et al*. [5] and 9–12 species found in two regions of the former German Democratic Republic respectively in 1966 and in 1980 [9]. Recent studies in the Northern European hemisphere [6,8] show species richness that fits the asymptotic maximum of the estimated species richness calculated from our data. This increase might be driven by a combination of natural developments and or anthropogenic causes (global warming, climatic fluctuations). It is however, beyond the scope of this paper to identify the drivers for the observed increase in the parasite biodiversity.

Parasites of veterinary importance may be introduced into the environment through pet travel or translocation of wildlife hosts. *Angiostrongylus vasorum* only recently became endemic to the Netherlands [41] and is known for its endemic foci in Dutch dogs [41]. In the present study, we found *A. vasorum*-positive foxes in the southern half of the study area, outside and distant from the published endemic foci, which demonstrates a wider endemic area sustained by the red fox.

In this study, *E. multilocularis* parasite DNA was identified by PCR in the intestinal content of one red fox in the northern part of the Dutch-German border area. The identification based solely on molecular techniques suggests a very low intestinal abundance in the infected fox, well below the detection level of microscopy. Previous studies showed PCR to be more sensitive, compared to the mucosal scraping method, especially at low endemicity [20,42].

The observed *T. canis* prevalence decline in foxes (−17%) is also recognised in the human population, since data from a Dutch cohort study show a moderate but significant decrease of *T. canis* exposure between 1998 and 2004 [43]. However, this is not recognised in prevalence of patent infections in dogs [44-47].

The prevalence of *Taenia* spp. showed the sharpest decline (−59%), followed by *T. canis* (−17%), compared to the study by Borgsteede [4]. Among fox prey are rodents, which are obligate intermediate hosts in the lifecycle of cestode parasites like *E. multilocularis* and *Taenia* spp., and facultative intermediate hosts of nematodes like *T. canis*. Small mammals, especially voles (*Microtus arvalis* and *Arvicola terrestris*), comprise almost 50% of the fox’s prey during autumn and winter [30,48,49]. The decreasing prevalence of *Taenia* spp. and *T. canis* in foxes might be correlated with the decreasing abundance of rodents [50,51], which is also indicated by decline of raptor species exclusively preying on rodents [52,53].

We were able to identify *Taenia crassiceps* and *T. polyacantha* from frozen material, using morphological data in combination with molecular techniques. A combination of detection techniques as presented in this study might be useful to increase sensitivity and specificity and to differentiate host-specific parasites from parasite eggs and/or larvae passing after ingestion of prey. CO1 gene sequences of *A. alata*, *T. crassiceps* and *T. polyacantha* from Dutch fox (this study) were homologous with isolates from European countries at the North or East of the Netherlands (Germany, Denmark, Lithuania, Finland and Russia). Previously, spatial prevalence analysis across borders demonstrated radiation of *E. multilocularis*, from the adjacent Belgian fox population to the southern Dutch fox population [20,54].

In conclusion, we infer a significant increase in parasitic helminths diversity in the fox population at the eastern border of the Netherlands over a period of 35 years. In the same period, the prevalence of two zoonotic helminths species belonging to different genera declined. In addition, four veterinary-important species were identified for the first time in this present study, and three additional species showed higher prevalence over that period. We identified the fox tapeworm *E. multilocularis* for the first time outside the previously described endemic spots in the Netherlands. Due to the very low prevalence and abundance, the infection risk for humans in the studied area is considered limited. It remains important, however, to follow the spread of *E. multilocularis* in this area in the future.

## Competing interests

The authors declare that they do not have competing interests.

## Authors’ contributions

FF generated and analysed parasitological data, performed molecular lab work and sequence analysis, and wrote the manuscript, RN generated parasitological data and wrote the manuscript, JM generated biological data concerning the collected foxes, HC generated parasitological data concerning non-intestinal helminths, CD did the molecular lab work concerning *E. multilocularis*, KT wrote the study design and manuscript, and helped with statistical analysis, JvdG wrote the study design, conceived and wrote the project proposal, coordinated the study, generated parasitological data and contributed to the manuscript. All authors read and approved the final manuscript.
